# Prevalence and susceptibility to antibiotics from *Campylobacter jejuni* and *Campylobacter coli* isolated from chicken meat in southern Benin, West Africa

**DOI:** 10.1186/s13104-020-05150-x

**Published:** 2020-06-26

**Authors:** Sylvain Daton Kouglenou, Alidehou Jerrold Agbankpe, Victorien Dougnon, Armando Djiyou Djeuda, Esther Deguenon, Marie Hidjo, Lamine Baba-Moussa, Honore Bankole

**Affiliations:** 1 Laboratory of Food Microbiology, Ministry of Health, 01, P.O. Box 418, Cotonou, Benin; 2 Research Unit in Applied Microbiology and Pharmacology of Natural Substances, Research Laboratory in Applied Biology, Polytechnic School of Abomey-Calavi, University of Abomey-Calavi, 01, P.O. Box 2009, Cotonou, Benin; 3 Laboratory for Public Health Research Biotechnologies, Biotechnology Center, University of Yaounde I, P.O. Box 812 Yaounde, Cameroon; 4 Laboratory of Biology and Molecular Typing in Microbiology, Faculty of Science and Technology, University of Abomey-Calavi, 05, P.O. Box 1604, Cotonou, Benin

**Keywords:** *Campylobacter jejuni*, *Campylobacter coli*, Chicken thighs, Antimicrobial resistance, Southern Benin

## Abstract

**Objective:**

Poultry is commonly considered to be the primary vehicle for Campylobacter infection in humans. The aim of this study is to assess the risk of Campylobacteriosis in chicken meat consumers in southern Benin by assessing the prevalence and resistance profile of *Campylobacter coli* and *Campylobacter jejuni* isolated from chicken thigh in Southern Benin.

**Results:**

The contamination rate of Campylobacter in the samples was 32.8%. From this percentage, 59.5% were local chicken thighs and 40.5% of imported chicken thighs (p = 0.045). After molecular identification, on the 256 samples analyzed, the prevalence of *C. jejuni* was 23.4% and 7.8% for *C. coli*, with a concordance of 0.693 (Kappa coefficient of concordance) with the results from phenotypic identification. Seventy-two-point seven percent of Campylobacter strains were resistant to Ciprofloxacin, 71.4% were resistant to Ampicillin and Tetracycline. 55.8% of the strains were multi-drug resistant.

## Introduction

Campylobacter, one of the food pathogens, has become one of the major causes of enteric infections in both developing and developed countries [1]. Incidence and prevalence of Campylobacteriosis has increased worldwide over the past decade, with approximately 500 million cases of gastroenteritis reported each year [2]. In Blantyre at Malawi, a 10-year study (1997–2007) found that *C. jejuni* and *C. coli* were detected in 21% (415/1941 children) of hospitalized children with diarrhea by real-time PCR, with *C. jejuni* accounting for 85% of all campylobacteriosis cases [3]. Campylobacter transmission mainly occurs following exposure to farm animals with such infections, with subsequent passage through the food chain to retail food products [4, 5]. Poultry are considered the main reservoir of infection and humans are most often infected by handling or consuming contaminated poultry meat [6].

Overuse or misuse of antimicrobials in the human population and in food animals has increased the number of antibiotic-resistant infections, including resistance to fluoroquinolones [7]. This resistance creates a real health problem, since the symptoms of campylobacteriosis are the same as those of gastrointestinal infections caused by other bacterial pathogens. Therefore, the empirical use of fluoroquinolones for the treatment of all gastrointestinal infections further promotes antibiotic resistance of this family [2]. In addition to the morbidity due to Campylobacteriosis and the risk of developing long-term sequelae, such as Guillian Barre syndrome (GBS), the development of antimicrobial resistance by Campylobacter strains constitutes an important concern [8].

A study in Benin reported contamination by *Campylobacter* spp. of imported poultry meat [9]. However, limited data exist on the health quality of local poultry meat about *Campylobacter* spp. In addition, it is important to know the resistance profile of these circulating Campylobacter strains in Benin. On this basis, we assessed the risk of Campylobacteriosis in chicken thighs consumers in southern Benin by determining the prevalence and susceptibility of Campylobacter strains isolated from samples of local and imported chicken meat commercialized in southern Benin.

## Main text

### Methods

#### Study framework

The sampling of chicken meat was carried out in the markets of the municipalities of Cotonou, Abomey-Calavi, Ouidah, Porto-Novo and Adjarra. All these markets are located in southern Benin. (Additional file 1: Figure S1). These communes were chosen because they belong to the three departments (Atlantique, Littoral and Oueme) which lead the pack when it comes to poultry farming in Benin [10].

#### Sampling

The sample size for this study was 256. It was estimated using Schwartz’s formula. The distribution of the different types of samples according to the markets and the municipalities is presented in Additional file 1: Table S1.

#### Bacteriological isolation

Samples were analyzed according to standard NF EN ISO 10272-1 modified [9, 11]. After enrichment and isolation on Karmali and Preston Campylobacter agars (at 42 °C in a microaerophilic atmosphere for 48 h), a characteristic Campylobacter colonies was seeded on nutritive agar enriched with fresh sheep blood and incubated in a microaerophilic atmosphere at 37 °C for 36 h. Pure cultures obtained were stored in Müller Hinton (MH) broth with glycerol (30%) at − 37 °C for additional analyzes.

The phenotypic identification of *Campylobacter* spp strains was carried out according to the methodology of Kougblenou et al. [11].

#### Identification of isolated Campylobacter strains by Polymerase Chain Reaction (PCR)

DNA of Campylobacter isolates were extracted using the Qiagen blue extraction kit. Molecular identification of isolates was carried out by PCR using *16SrRNA* (816 bp) primer specific to all species Campylobacter: C412F 5′-GGATGACACTTTTCGGAGC-3′ and C1228R 5′-CATTGTAGCACGTGTGTC-3′ [12, 13]. Then, the isolates were identified as *C. jejuni* and *C. coli* using specific primers. *C. jejuni* (*mepA* (413 bp)): CJmapAN3F 5′-TGGTGGTTTTGAAGCAAAGA-3 ‘and CJmapAN3R 5′-GCTTGGTGCGGATTGTAAA-3′ [11, 13]; *C. coli* (*ceuE* (330 bp)): CCceuEN3F 5′-AAGCGTTGCAAAACTTTATGG-3′and CCceuEN3R’ 5-CCTTGTGCGCGTTCTTTATT-3′ [12, 14]. The PCR products was run on gel electrophoresis. For each PCR reaction, two positive controls were carried out using the reference strains *C. jejuni* ATCC 29428 and *C. coli* ATCC 33559.

#### Antibiotic susceptibility testing of Campylobacter isolates

The determination of the sensitivity to antibiotics was carried out on all strains isolated, according to the method of diffusion on disc [15]. The choice of the 6 antibiotics tested (Additional file 1: Table S2) is justified by the fact that, the drugs of choice used in the clinical therapy of campylobacteriosis are macrolides, quinolones, fluoroquinolones and tetracyclines [15–18].

#### Statistical analysis

Data were analyzed with statistical software R version 3.6.1. The difference was significant when p < 0.05. In addition, a 95% confidence interval (95% CI) was also determined for antibiotic resistance rates.

### Results

#### Results of bacteriological culture of samples

32.8% of all chicken thigh samples analyzed tested positive for Campylobacter culture. Of the 84 positive samples, 50 (59.5%) were local chicken thighs and 34 (40.5%) were imported chicken thighs, representing respectively 39.1% of the samples from local chicken thighs and 26.6% of imported chicken thigh samples (Additional file 1: Table S3). This difference in frequency of positivity is statistically significant (p = 0.045) between the samples of local and imported chicken thighs.

#### Campylobacter isolation

The PCR results showed that out of all the 80 phenotypically identified Campylobacter strains (*C. jejuni* and *C. coli*), the identity of 77 were confirmed. Of the 77 confirmed strains, 45 were isolated from local chicken thighs and 32 were from imported chicken thighs. Of the 60 strains of *C. jejuni* obtained after phenotypic identification 55 were confirmed *C. jejuni*, 2 strains were identified *C. coli* and three strains which are neither *C. jejuni* nor *C. coli*. That is to say 91.7% of identity match of the strains of *C. jejuni*. As for the 20 phenotypically identified *C. coli* strains, 14 were confirmed *C. coli* and 6 *C. jejuni* by PCR, i.e. 70% of identity match of the *C. coli* strains (Table 1 and Additional file 1: Figure S2). Analysis of these results showed that there is a concordance between the results of the phenotypic identification and those of the molecular identification with the coefficient of kappa which is equal to 0.693.

**Table 1:** Comparison of identification of Campylobacter species by PCR with that obtained phenotypically

Municipalities	Type of samples n (%)							
	Local chicken thighs				Imported chicken thighs			
	Phenotypic identification		Identification by PCR		Phenotypic identification		Identification by PCR	
	*C. jejuni*	*C. coli*	*C. jejuni*	*C. coli*	*C. jejuni*	*C. coli*	*C. jejuni*	*C. coli*
Abomey-Calavi	8 (24.2)	4 (28.6)	8 (23.5)	4 (36.4)	8 (29.6)	0	7 (26.0)	0
Adjarra	3 (9.1)	3 (21.4)	5 (14.7)	1 (9.1)	5 (18.5)	1 (16.7)	5 (18.5)	1 (20.0)
Cotonou	8 (24.2)	2 (14.3)	6 (17.6)	2 (18.2)	4 (14.8)	2 (33.3)	4 (14.8)	2 (40.0)
Ouidah	5 (15.2)	1 (7.1)	6 (17.6)	0	4 (14.8)	2 (33.3)	4 (14.8)	2 (40.0)
Porto-Novo	9 (27.3)	4 (28.6)	9 (26.5)	4 (36.4)	6 (22.2)	1 (16.7)	7 (26.0)	0
Total	33	14	34	11	27	6	27	5

#### Antibiotic resistance of identified Campylobacter strains

The antibiotic susceptibility test was carried out on the 77 strains whose species level identities were confirmed by PCR. 72.7% of these strains were resistant to ciprofloxacin, 71.4% were resistant to ampicillin and tetracycline respectively, 19.5% of strains showed resistance to amoxicillin + clavulanic acid, 11.7% showed resistance to erythromycin and 7.8% showed resistance to gentamicin (Table 2). Analysis of the results showed that the difference in resistance percentages between the strains of *C. jejuni* and *C. coli* with ampicillin (p = 0.001) and tetracycline (p = 0.030) is statistically significant. The strains of *C. jejuni* and *C. coli* did not show strong resistance to erythromycin (*C. jejuni* (4.9%); *C. coli* (37.5%); 95% CI = 2.0–79.6), but the difference in their resistance percentages was statistically significant (p = 0.001).

**Table 2:** Susceptibility of *C. jejuni* and *C. coli* strains identified to the different antibiotics used

Families of antibiotics used	Antibiotics used	All strains identified n = 77 (%)	Type of samples			Campylobacter species identified		
			Local chicken thighs n = 45 (%)	Imported chicken thighs n = 32 (%)	95% CI^b^	*C. jejuni* n = 61 (%)	*C. coli* n = 16 (%)	95% CI^b^
β-Lactams	AMC	15 (19.5)	10 (22.2)	5 (15.6)	0.4–6.4	12 (19.7)	3 (18.8)	0.1–4.3
	AMP	55 (71.4)	31 (68.9)	24 (75.0)	0.2–2.3	49^a^ (80.3)	6^a^ (37.5)	0.0–0.6
Fluoroquinolones	CIP	56 (72.7)	35 (77.8)	21 (65.6)	0.6–5.7	42 (68.9)	14 (87.5)	0.6–31.1
Macrolides	E	9 (11.7)	6 (13.3)	3 (9.4)	0.3–10.0	3^a^ (4.9)	6^a^ (37.5)	2.0–79.6
Tetracycline	TE	55 (71.4)	33 (73.3)	22 (68.8)	0.4–3.8	40^a^ (65.6)	15^a^ (93.8)	1.0–346.5
Aminoglycosides	GM	6 (7.8)	3 (6.7)	3 (9.4)	0.1–5.5	4 (6.6)	2 (12.5)	0.2–15.8

*AMC* Amoxicillin + Clavulanic Acid, *AMP* Ampicillin, *CIP* Ciprofloxacin; Erythromycin, *TE* Tetracycline, *GM* Gentamicin

^a^Statistically significant difference between the two proportions

^b^95% Confidence interval

The distribution of antibiotic resistance among Campylobacter strains has shown that the number of antibiotic resistant strains differs from one municipality to another, depending on the antibiotic disc (Additional file 1: Figure S3). This discrepancy in the level of antibiotic resistance is statistically significant between the five municipalities with regard to amoxicillin + clavulanic acid (p = 0.015), ampicillin (p = 0.001) and gentamicin (p = 0.016).

With regard to the multidrug resistance of the isolated strains, 43 (55.8%) strains of Campylobacter were resistant to at least 3 antibiotics belonging to three different families. 26 (60.5%) of these strains were resistant to 3 antibiotics, 13 (30.2%) were resistant to 4 antibiotics, 3 (7%) to 5 antibiotics and one (2.3%) to 6 antibiotics (Table 3).

**Table 3:** Multi-resistance profiles of the strains identified according to the type of sample and the Campylobacter species

Number of antibiotics from different families	Multi-resistance profiles	All strains identified n = 77 (%)	Type of samples		Campylobacter species identified	
			Local chicken thighs n = 45 (%)	Imported chicken thighs n = 32 (%)	*C. jejuni* n = 61	*C. coli* n = 16
3	*AMP*-*CIP*-*TE*	21 (48.8)	11 (40.7)	10 (62.5)	21 (60.0)	0
	*AMC*-*AMP*-*TE*	2 (4.7)	1 (3.7)	1 (6.3)	2 (5.7)	0
	*CIP*-*E*-*TE*	1 (2.3)	1 (3.7)	0	0	1 (12.5)
	*AMC*-*CIP*-*TE*	1 (2.3)	1 (3.7)	0	0	1 (12.5)
	*AMC*-*AMP*-*CIP*	1 (2.3)	1 (3.7)	0	1 (2.9)	0
4	*AMC*-*AMP*-*CIP*-*TE*	5 (11.6)	4 (14.8)	1 (6.3)	5 (14.3)	0
	*AMP*-*CIP*-*E*-*TE*	6 (14.0)	5 (18.5)	1 (6.3)	2 (5.7)	4 (50.0)
	*AMP*-*CIP*-*TE*-*GM*	1 (2.3)	0	1 (6.3)	1 (2.9)	0
	*AMC*-*AMP*-*CIP*-*GM*	1 (2.3)	0	1 (6.3)	1 (2.9)	0
5	*AMC*-*AMP*-*CIP*-*TE*-*GM*	2 (2.6)	2 (7.4)	0	1 (2.9)	1 (12.5)
	*AMC*-*AMP*-*CIP*-*E*-*TE*	1 (2.3)	0	1 (6.3)	1 (2.9)	0 (12.5)
6	*AMC*-*AMP*-*CIP*-*E*-*TE*-*GM*	1 (2.3)	1 (3.7)	0	0	1 (12.5)
Total		43 (55.8)	27 (60.0)	16 (50.0)	35 (57.4)	8 (50.0)

*AMC* Amoxicillin + Clavulanic Acid, *AMP* Ampicillin, *CIP* Ciprofloxacin; Erythromycin, *TE* Tetracycline, *GM* Gentamicin

## Discussion

Out of the 256 chicken thigh samples analyzed, 84 samples were positive for *Campylobacter* spp culture, representing a 32.8% contamination rate. In the five municipalities involved in this study, these rates are 40.6% in Adjarra, 32.8% in Abomey-Calavi and Porto-Novo, 37.5% in Ouidah and 26.6% in Cotonou (Additional file 1: Table S3). Although these contamination rates are different from one municipality to another, there is no statistically significant difference between the level of contamination in these municipalities (p > 0.05). This means that consumers of chicken meat from any of the municipalities involved in this study are almost similarly exposed to the risk of Campylobacter foodborne infections. Moreover, the obtained results have shown a statistically significant lower contamination rate of imported chicken thighs (26.6%) compared to the local ones (39.1%) (p = 0.045). These results are in agreement with those found by [9] in Benin, where the prevalence of contamination of poultry meat imported into Benin was 20%. The high contamination rate of local chicken meat compared to that of imported meat, shows that there is a lack of hygiene during the production of local chicken meat. Thus, Benin has not yet reached the level of hygiene recommended by the World Health Organization and the joint committee of the Food and Agriculture Organization of the United Nations, when handling products meat [19].

In the present study, the prevalence is 23.8% for *C. jejuni* and 6.3% for *C. coli* in chicken meat in southern Benin. This prevalence is low, but higher than those obtained in the spring (11% for *C. jejuni*, 0% for *C. coli*), in summer (11% for *C. jejuni*, 0% for *C. coli*) and in winter (2.5% for *C jejuni*, 1% for *C. coli*) in broilers in Tunisia [8]. The strains of *C. jejuni* (55.7%) and *C. coli* (68.8%) were found mostly in the samples of local chicken thighs. These results are in agreement with those found by [20], where the prevalence of *C. jejuni* (46.1%) and *C. coli* (32.8%) in poultry meat in Korea is very high than that obtained in poultry meat imported into the country.

Several studies have shown resistance of Campylobacter strains to fluoroquinolones, tetracyclines and macrolides [2, 17, 21–25]. The susceptibility of Campylobacter strains isolated in the present study showed 72.7% resistance to fluoroquinolones (Ciprofloxacin), 71.4% resistance to tetracyclines (Tetracycline) and β-lactams (Ampicillin). The high level of resistance of the strains to these three antibiotics was observed in strains isolated both from local and imported chicken thighs. High resistance rate of Campylobacter strains to these three antibiotics has also been observed in Algeria on Campylobacter strains isolated from turkeys [24]. In northern Tunisia, Campylobacter strains isolated from chicken meat samples showed strong resistance to ampicillin (61.4%), ciprofloxacin (99.2%) and tetracycline (100%) [8]. These results can be explained by the common and sometimes uncontrolled use of the same antibiotics in poultry farms to fight against bacterial infections on farms. Indeed, a clear association between the use of fluoroquinolones and tetracyclines in poultry production and the high resistance rate of Campylobacter isolated from poultry has been shown by several studies [26–28]. The strains of *C. jejuni* are more resistant to ampicillin than those of *C. coli*. While resistance to tetracycline and erythromycin is more observed in strains of *C. coli* than in strains of *C. jejuni*. These results are in agreement with those found by [17] and [4].

More than half (55.8%) of the isolated Campylobacter strains were multidrug-resistant with at least three antibiotics belonging to three different families and twelve determined multidrug resistance profiles. These data show how often the emergence of multidrug resistance in bacterial strains is increasing in Benin. Such alarming percentages of multidrug resistance of Campylobacter strains were also observed in other countries, 96.6% in Algeria [24], 90% in South Africa [29] and 69% in Poland [17]. The AMP-CIP-TE resistance profile was most observed in the strains of *C. jejuni* (52.5%). This pattern has been observed with similar frequency in Korea with strains of *C. jejuni* isolated from poultry meat samples [20]. Molecular characterization of resistance genes is imperative for a better understanding of gene transmission and the mechanisms behind these resistances.

## Conclusion

Results from this study show that there is a real risk of Campylobacter poisoning among consumers of chicken meat in southern Benin. In addition, these isolated Campylobacter strains are multidrug-resistant, which poses a problem in selecting Campylobacter strains in chicken farms, where antibiotics are used in an anarchic manner.

## Limitations

Absence of characterization of resistance genes, and sequencing of Campylobacter genome constitutes the limit of this study.

## Supplementary information

**Additional file 1: Figure S1.** Map of southern Benin showing the study area and sampling sites; **Table S1.** Distribution of different types of samples; **Table S2.** List of antibiotics tested and their respective loads; **Table S3.** Distribution of culture results according to the nature of the samples, markets and municipalities where the samples were taken; **Figure S2.** Photo of agarose gel electrophoresis of amplicons of some Campylobacter strains isolated from chicken thighs; **Figure S3.** Frequency of resistance of Campylobacter strains as a function of the sampling area.

## Data Availability

All data generated or analysed during this study is included in this published article and Additional file.

## Abbreviations

GBS: Guillian Barre Syndrome
AMP: Ampicillin
GM: Gentamicin
E: Erythromycin
CIP: Ciprofloxacin
TE: Tetracycline
AMC: Amoxicillin + clavulanic acid
CI: Confidence interval
PCR: Polymerase Chain Reaction
MH: Müller Hinton
