# Supplementary material for: Prevalence and susceptibility to antibiotics from Campylobacter jejuni and Campylobacter coli isolated from chicken meat in southern Benin, West Africa
Source: BMC Res Notes. 2020 Jun 26;13:305. doi: 10.1186/s13104-020-05150-x (PMC7318530; doi:10.1186/s13104-020-05150-x)
Supplement: Supplementary file 1 — Additional file 1: Figure S1. Map of southern Benin showing the study area and sampling sites; Table S1. Distribution of different types of samples; Table S2. List of antibiotics tested and their respective loads; Table S3. Distribution of culture results according to the nature of the samples, markets and municipalities where the samples were taken; Figure S2. Photo of agarose gel electrophoresis of amplicons of some Campylobacter strains isolated from chicken thighs; Figure S3. Frequency of resistance of Campylobacter strains as a function of the sampling area. [file 13104_2020_5150_MOESM1_ESM.docx]

**Additional file 1**


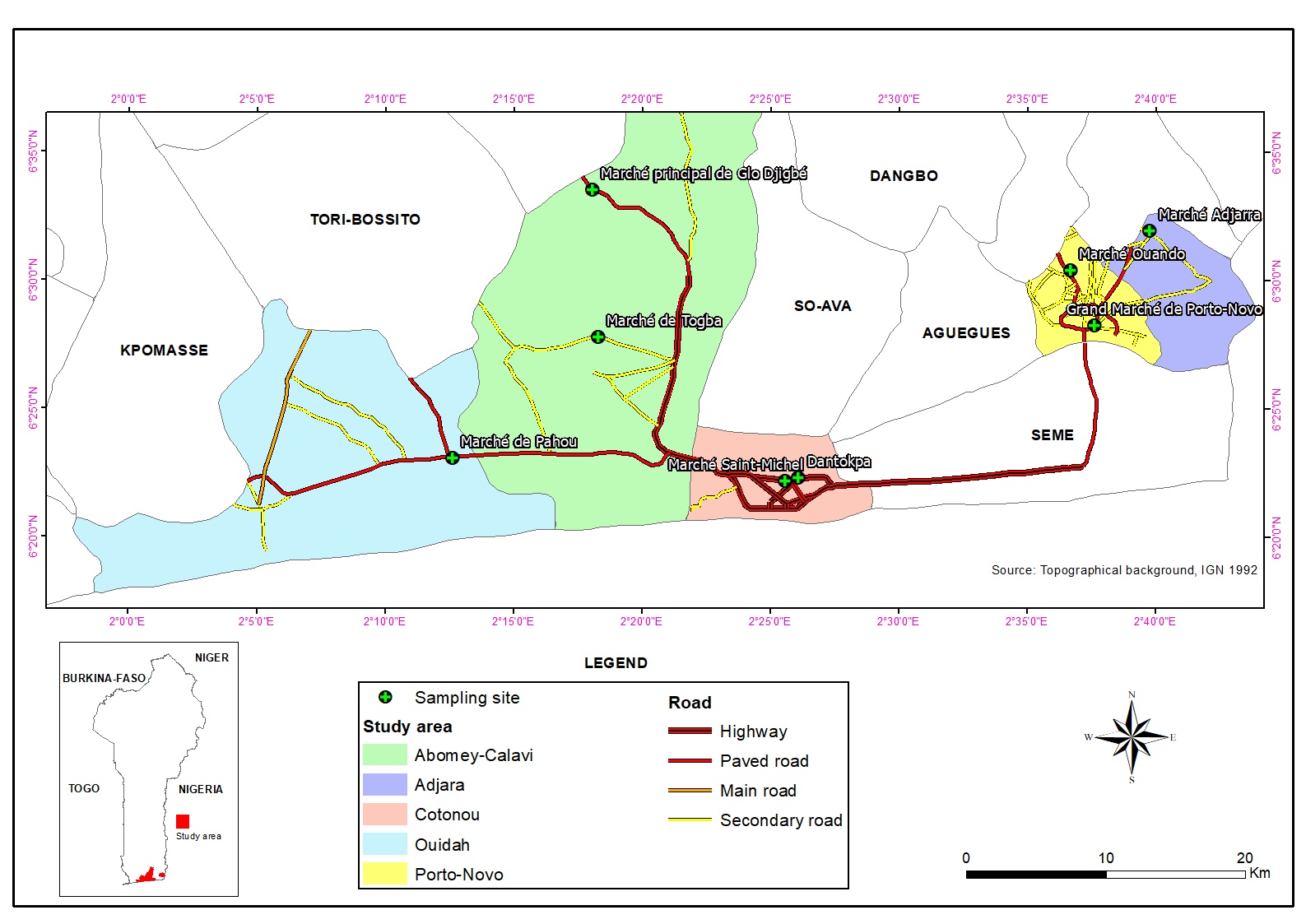


**Figure S1**. Map of southern Benin showing the study area and sampling sites

**Table S1.** Distribution of different types of samples

| Municipalities | Markets | Type of samples | Effectives | Total |
| --- | --- | --- | --- | --- |
| Cotonou | C1 | Local chicken thighs | 16 | **64** |
|  |  | Imported chicken thighs | 16 |  |
|  | C2 | Local chicken thighs | 16 |  |
|  |  | Imported chicken thighs | 16 |  |
| Abomey-Calavi | A1 | Local chicken thighs | 16 | **64** |
|  |  | Imported chicken thighs | 16 |  |
|  | A2 | Local chicken thighs | 16 |  |
|  |  | Imported chicken thighs | 16 |  |
| Porto-Novo | P1 | Local chicken thighs | 16 | **64** |
|  |  | Imported chicken thighs | 16 |  |
|  | P2 | Local chicken thighs | 16 |  |
|  |  | Imported chicken thighs | 16 |  |
| Ouidah | O | Local chicken thighs | 16 | **32** |
|  |  | Imported chicken thighs | 16 |  |
| Adjarra | Ad | Local chicken thighs | 16 | **32** |
|  |  | Imported chicken thighs | 16 |  |
| Total | | | | **256** |

C1 : Dantokpa ; C2 : Saint Michel ; A1 : Togba ; A2 : Glodjigbe ; P1 : Ouando ; P2 : Grand-Marche ; O : Pahou ; Ad : Ajarra market

**Table S2.** List of antibiotics tested and their respective loads

| Antibiotics tested | | Loads (µg) |
| --- | --- | --- |
| Ampicillin | (AMP) | 10 |
| Gentamicin | (GM) | 10 |
| Erythromycin | (E) | 15 |
| Ciprofloxacin | (CIP) | 5 |
| Tetracycline | (TE) | 30 |
| Amoxicillin + Clavulanic acid | (AMC) | 20 |

**Table S3.** Distribution of culture results according to the nature of the samples, markets and

municipalities where the samples were taken.

| Municipalities | Markets | Culture results  n (%) | | | | | | Total number of samples |
| --- | --- | --- | --- | --- | --- | --- | --- | --- |
|  |  | **Local chicken thighs**  **n = 16 / market** | | **Imported chicken thighs**  **n = 16 / market** | | **Total** | |  |
|  |  | **-** | **+** | **-** | **+** | **-** | **+** |  |
| Cotonou | C1 | 10 (62.1) | 6 (37.5) | 12 (75.0) | 4 (25.0) | 22 (68.8) | 10 (31.2) | **32** |
|  | C2 | 12 (75.0) | 4 (25.0) | 13 (81.2) | 3 (18.8) | 25 (78.1) | 7 (21.9) | **32** |
|  | **Total** | **22 (68.8)** | **10 (31.2)** | **25 (78.1)** | **7 (21.9)** | **47 (73.4)** | **17 (26.6)** | **64** |
| Abomey-Calavi | A1 | 10 (62.1) | 6 (37.5) | 11 (68.7) | 5 (31.3) | 21 (65.6) | 11 (34.4) | **32** |
|  | A2 | 9 (56.3) | 7 (43.7) | 13 (81.2) | 3 (18.8) | 22 (68.8) | 10 (31.2) | **32** |
|  | **Total** | **19 (59.4)** | **13 (40.6)** | **24 (75.0)** | **8 (25.0)** | **43 (67.2)** | **21 (32.8)** | **64** |
| Porto-Novo | P1 | 9 (56.2) | 7 (43.8) | 12 (75.0) | 4 (25.0) | 21 (65.6) | 11 (34.4) | **32** |
|  | P2 | 9 (56.2) | 7 (43.8) | 13 (81.2) | 3 (18.8) | 22 (68.8) | 10 (31.2) | **32** |
|  | **Total** | **18 (56.2)** | **14 (43.8)** | **25 (78.1)** | **7 (21.9)** | **43 (67.2)** | **21 (32.8)** | **64** |
| Ouidah | O | 10 (62.1) | 6 (37.5) | 10 (62.1) | 6 (37.5) | **20 (62.5)** | **12 (37.5)** | **32** |
| Adjarra | Ad | 9 (56.2) | 7 (43.8) | 10 (62.1) | 6 (37.5) | **19 (59.4)** | **13 (40.6)** | **32** |
| Total | **-** | **78 (60.9)** | **50 (39.1)** | **94 (73.4)** | **34 (26.6)** | **172 (67.2)** | **84 (32.8)** | **256** |

(-) : culture negative samples ; (+) : culture positive samples ; n : effective


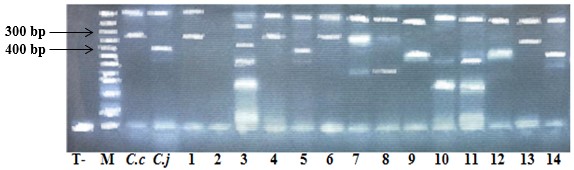


Legendre: *C.c* = *Campylobacter coli* ATCC; *C.j* = *Campylobacter jejuni* ATCC 29428; 1-4-6-7-13 = identified *Campylobacter coli* strains; 5-9-12-14 = identified *Campylobacter jejuni* strains

**Figure S2**. Photo of agarose gel electrophoresis of amplicons of some Campylobacter strains isolated from chicken thighs


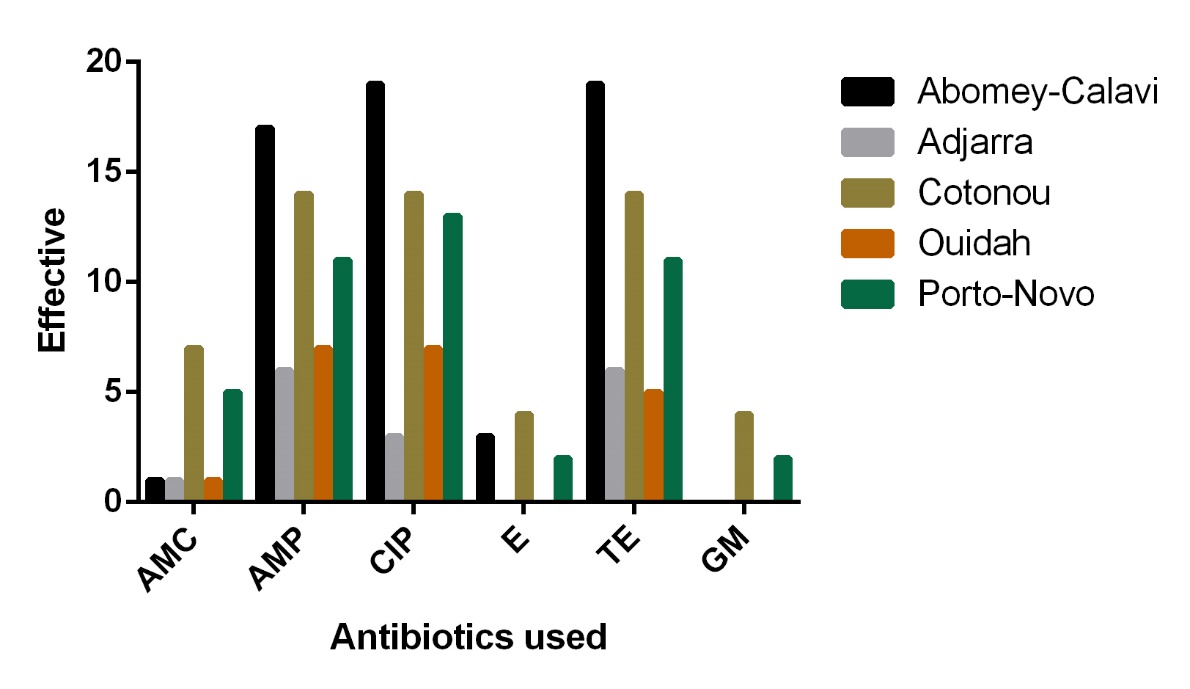


Legendre = AMC: Amoxicillin + Clavulanic Acid; AMP: Ampicillin; CIP: Ciprofloxacin; Erythromycin; TE: Tetracycline; GM: Gentamicin

**Figure S3.** Frequency of resistance of Campylobacter strains as a function of the sampling area
